# A study on willingness to take the COVID-19 vaccine at a tertiary institution community in Johannesburg, South Africa

**DOI:** 10.4102/phcfm.v14i1.3252

**Published:** 2022-07-28

**Authors:** Bhadrashil H. Modi, Deidré Pretorius, Joel M. Francis

**Affiliations:** 1 Division of Family Medicine, Department of Family Medicine and Primary Care, School of Clinical Medicine, University of the Witwatersrand, Johannesburg, South Africa; 2 College of Medicine, Cape Town, South Africa; 3 Private Practice Family Physician, Johannesburg, South Africa; 4 Department of Family Medicine and Primary Care, School of Clinical Medicine, University of the Witwatersrand, Johannesburg, South Africa

**Keywords:** COVID-19, South Africa, vaccination, vaccine hesitancy, willingness to be vaccinated

## Abstract

**Background:**

South Africa is aiming to achieve herd immunity against the coronavirus disease 2019 (COVID-19) by the first quarter of 2022. The success of the COVID-19 vaccination roll-out depends primarily on the willingness of the population to take the vaccines.

**Aim:**

This study aimed to examine the willingness to take the COVID-19 vaccine, along with the factors of concern, efficacy and preferences of the individual, which may increase the willingness to be vaccinated.

**Setting:**

This study was conducted at the University of the Witwatersrand, Johannesburg, amongst adult students and academic and professional staff.

**Methods:**

A cross-sectional online survey from 27 July 2021 to 14 August 2021 was conducted. We performed descriptive and inferential analysis to determine the factors associated with willingness to take the COVID-19 vaccine.

**Results:**

A total of 2364 participants responded to a survey link and 82.0% were students, 66.8% were in the 18–29 years age band and females represented 64.0%. A total of 1965 participants (83.3%) were willing to receive a COVID-19 vaccine, the most preferred vaccines were Pfizer (41%) and J&J (23%), local pharmacy (29%) and General Practitioner (GP) (17%) were the preferred places for vaccination and the trusted sources of information on COVID-19 vaccines were the general practitioners (40.6%) and specialists (19.2%). Perceptions that vaccines are safe (adjusted odds ratio [aOR] = 31.56, 95% confidence interval [CI]: 16.02–62.12 for affirmative agreement) and effective (aOR = 5.92, 95% CI: 2.87–12.19 for affirmative agreement) were the main determinants of willingness to taking a COVID-19 vaccine.

**Conclusion:**

It is imperative to reinforce the message of COVID-19 vaccine safety and efficacy and to include the GPs and the community pharmacies in the vaccination roll-out in South Africa.

## Background

Vaccination for the coronavirus disease 2019 (COVID-19) is the primary intervention, together with the other public health measures to curb the COVID-19 pandemic.^1^ The key success factor of the COVID-19 vaccination programme is the willingness to be vaccinated, in addition to other factors such as effective communication and accessibility to the vaccines.^2^ The willingness to be vaccinated also influences the speed of the vaccination programme, which is crucial as each vaccine administered implies fewer COVID-19 cases, hospitalisation and death.^3^

By mid-August 2021, 190 countries have begun mass vaccination campaigns and 4.7 billion doses of the COVID-19 vaccines have been administered worldwide.^4^ In Europe and North America, where a large number of people have been vaccinated, there is a positive effect on reduced mortality, improved consumer, and business confidence and thus an improvement of the overall economy due to the opening of the countries.^5^ South Africa (SA) is aiming to vaccinate approximately 40 million adults over the age of 18 years (67% of the population) to achieve herd immunity.^6^ To achieve this herd immunity by the first quarter of 2022, SA aims to vaccinate more than 200 000 people daily.^7^

A global Independent Polling System of Society^8^ (IPSOS) survey undertaken in January 2021 in 15 countries showed that the population in the United Kingdom had the highest intent on being vaccinated (89%) and the lowest was in Russia (42%). The South African results showed a 61% intent on being vaccinated.^9^

Evidence suggests that conspiracy beliefs and misinformation increase vaccine hesitancy and decrease the willingness to be vaccinated.^10^ It’s been reported that people who believe in such misinformation are four times less likely to be willing to be vaccinated and more likely to trust the information on social media.^10^

The Wellcome Global Monitor 2018^11^ reported that most people think vaccines are safe and effective. They further reported that almost 75% of the people, when it comes to health advice, would trust a doctor or nurse over anyone else (family, friends, leaders or famous people) in deciding to be vaccinated or not. The Kaiser Family Fund (KFF) COVID-19 vaccine monitor^12^ survey found that most people in the United States, turned to doctors, nurses and other healthcare providers when deciding to be vaccinated. A follow-up survey found that 75% of people wish to obtain their COVID-19 vaccine at their doctor’s office, with 15% of the people preferring pharmacies, followed by a hospital (9%).^13^ Trust relationships exist not only in interpersonal relationships, such as the doctor–patient relationship but also with institutions. A trust-based relationship occurs when there is vulnerability on part of the truster because of a knowledge imbalance and a lack of power within health institutions and the importance of one’s health and well-being.^14^

There is limited evidence concerning the people’s willingness to take the COVID-19 vaccine in resource-constrained countries. In this study, we examined the willingness to take the COVID-19 vaccine, along with the factors of concern, efficacy and preferences of the individual that may increase the willingness to be vaccinated.

## Methods

### Study design

The study was a cross-sectional online survey of adult students and staff members at the University of the Witwatersrand, Johannesburg, South Africa.

### Study population and setting

The study population was adults studying or working at the University of the Witwatersrand. All students, academicians and the administrative and service staff older than 18 years were eligible to participate in the study. This university has a campus in Braamfontein and Parktown with approximately 40 129 registered students, 1642 academic staff members and nearly 3960 administrative and service staff members.

### Sample size and sampling

Using the OpenEpi software^15^ the sample was calculated based on the following assumptions, Wits community (students, academicians, administrative and service staff) population (*N* = 42 000), 50% of the participants will be willing to undertake COVID-19 vaccine, marginal error 5%, design effect of 2 and the 70% online survey non-response rate resulted in the estimated minimum sample size of 1300. We expected the distribution of participants to be at the ratio of 9:1 (1170 students and 130 staff). All adult students and staff were invited to participate in the study.

### Data collection procedures and ethical considerations

After permission was obtained from the University of the Witwatersrand registrar, the registrar’s office sent the survey link to all students and staff. The email contained information on the study and participants had to consent before they could access the survey questionnaire. The questionnaire was based on the number of factors, which includes the acceptability of the COVID-19 vaccine,^16^ the influence of social media,^17^ personal preferences^13^ and accessibility and convenient sites that feels safe.^18^ The questionnaire had theoretical and face validity. The online survey ran from 27 July 2021 to 14 August 2021 and was closed thereafter. The survey responses were captured in Research Electronic Data Capture (REDCap) database.^19^ The data were stored in a password-protected cloud server. No identifying information was collected from the participants.

### Data management and analysis

Data from REDCap was exported to Stata version 14 for management and analysis. The data set contained categorical variables and therefore the descriptive statistics frequencies (proportion) is summarised. The Chi-square test was used to determine the bivariate association of factors associated with willingness to undertake the COVID-19 vaccine. It was decided *a priori* that age and gender will be forced in the multivariable logistic regression model regardless of the bivariate analysis. The *p*-value and other factors were entered into the logistic regression multivariable model if the bivariate analysis *p*-value was < 0.20. The authors reported adjusted odds ratios (aORs) and considered a *p*-value of < 0.05 statistically significant.

### Ethical considerations

Approval to conduct the study was received from the Human Research Ethics Committee (Medical) of the University of the Witwatersrand (M210495).

## Results

### General characteristics of the study population

The university registrar shared the survey link with 40 129 students, 1642 academic staff and 3960 professional and administrative staff. The survey response rate was 2364/45 731 (5.0%). Table 1 shows that almost all participants responded to all survey questions 2330 (98.6%). Amongst all participants (*n* = 2364), 82.0% were students, 66.8% were in the 18–29 years age band and females represented 64.0% of all participants.

**TABLE 1:** General characteristics of the study participants (*N* = 2364).

Characteristic	Categories	*n*	%
Completeness of the responses	Incomplete	34	1.4
	Complete	2330	98.6
	Total	2364	100.0
Age (years)	18–29	1580	66.8
	30–49	571	24.2
	50 and above	213	9.0
	Total	2364	100.0
Gender	Male	829	35.1
	Female	1514	64.0
	Other	21	0.9
	Total	2364	100.0
Highest level of education	Up to grade 12	732	31.0
	Diploma and undergraduate degree	761	32.2
	Postgraduate degree	867	36.7
	Total	2360	100.0
Status (position) at the university	Student	1936	82.0
	Professional staff	138	5.8
	Academic staff	236	10.0
	Services	50	2.1
	Total	2360	100

### Willingness to receive COVID-19 vaccines and the preferred vaccines type

Table 2 further shows that 395/2360 participants (16.7%) indicated that they do not wish to receive COVID-19 vaccines. Amongst the 83.3% who indicated their willingness to take the COVID-19 vaccines – 30.7% had vaccinated (fully or partially). The most preferred COVID-19 vaccine types by the participants were Pfizer (41.4%) and J&J (23.3%) vaccines. The least preferred ones were Sinovac (0.8%), AstraZeneca (1.9%) and Sputnik (1.8%). Amongst those willing to take the vaccines their main motivations were concern about their personal well-being (60.4%) and concerns for the well-being of others (61.3%). Side effects (58.0%) and short timelines of the vaccine trials (34.4%) were major concerns of the COVID-19 vaccines reported by the participants.

**TABLE 2:** Perceptions and willingness to take the COVID-19 vaccines.

Characteristic	Categories	*n*	%
COVID-19 vaccines are safe	Agree	1134	48.1
	Somewhat agree	842	35.7
	Somewhat disagree	226	9.6
	Disagree	157	6.7
	Total	2359	100.0
COVID-19 vaccines are effective	Agree	1459	61.9
	Somewhat agree	670	28.4
	Somewhat disagree	121	5.1
	Disagree	107	4.5
	Total	2357	100.0
What is your COVID-19 vaccination status	Not eligible as yet	969	41.1
	Registered, but not vaccinated	272	11.5
	Vaccinated – partially	302	12.8
	Vaccinated – fully	422	17.9
	Do not wish to be vaccinated	395	16.7
	Total	2360	100.0
What is your preferred COVID-19 vaccine	J & J	549	23.3
	Pfizer	977	41.4
	Sputnik	42	1.8
	Astra Zeneca	45	1.9
	Sinovac	18	0.8
	Does not matter which one	430	18.2
	None	298	12.6
	Total	2359	100.0
Concerns about COVID-19 vaccines (*n* = 2364)†	No concern	722	30.5
	Worried about side effects	1372	58.0
	Vaccine trials were too fast	813	34.4
	Risk of getting COVID-19 is low	140	5.9
	Vaccines are not effective	311	13.2
	I am against vaccines in general	92	3.9
	Other reasons	190	8.0
	Total	2364	-
Reasons for taking the COVID-19 vaccine (*n* = 2364)†	Will not take the vaccine	324	13.7
	Concerned of personal well-being	1428	60.4
	Concerned of well-being of others	1450	61.3
	It is the right thing to do	1141	48.3
	Fear of dying	504	21.3
	Travelling	767	32.4
	Pressure from employer	152	6.4
	Other reasons	110	4.7

COVID-19, coronavirus disease 2019; J&J, Johnson and Johnson.

† , Each category was a standalone question.

### Perceptions on safety and effectiveness of the COVID-19 vaccines

Of the participants (*n* = 2359) who responded to the question on the safety of COVID-19 vaccines, 1134 (48.1%) agreed affirmatively that the vaccines were safe and 35.7% of the respondents somewhat agreed that the vaccines were safe. On the other hand, 2357 participants who responded to the vaccine effectiveness question, 1459 (61.9%) agreed affirmatively that the COVID-19 vaccines were effective and 28.4% indicated that they somewhat agreed (Table 2).

### Preferred COVID-19 vaccination places and the sources of COVID-19 information

Table 3 describes the preferred places for vaccination and sources of information. Participant’s preferences were a local pharmacy (28.8%), general practitioner (17.2%), hospital (9.9%), local health clinic (8.3%), workplace (7.7%) and vaccination site run by the government (7.8%). The trusted sources of information on COVID-19 vaccines were the general practitioners (40.6%), specialists (19.2%), the Government Department of Health (12.6%) and the medical aids (6.7%). The participants of this study postulated that the main sources of information were by the Government Department of Health (60.3%), via mainstream media (Radio, television, newspapers) (56.4%), social media (48.4%) and by medical aids (26.0%).

**TABLE 3:** Preferred vaccination place and sources of information for COVID-19 vaccines.

Characteristic	Categories	*n*	%
Most preferred vaccination place	Local pharmacy	679	28.8
	General practitioner	405	17.2
	Hospital	233	9.9
	Local health clinic	195	8.3
	Workplace	181	7.7
	Vaccination site run by the government	185	7.8
	Local school	97	4.1
	Local church or religious centre	23	1.0
	Traditional healer	2	0.1
	Other places	68	2.9
	Not applicable	292	12.4
	Total	2360	100.0
Most trusted people/organisations	Family or friends	108	4.6
	Traditional healer	21	0.9
	Religious leader	40	1.7
	General practitioner	958	40.6
	Specialist	453	19.2
	Nurse	48	2.0
	Pharmacist	63	2.7
	Famous/prominent person	7	0.3
	Government/Department of Health	296	12.6
	Employer	11	0.5
	Medical aid	157	6.7
	Other sources	195	8.3
	Total	2357	100.0
Sources of information (*n* = 2364)†	Social media – Facebook, Twitter	1145	48.4
	Other media – Radio, television, newspaper	1333	56.4
	Friends and family	726	30.7
	Government/Department of Health	1425	60.3
	Medical aid	615	26.0
	General practitioner	149	6.3
	Pharmacist	11	0.5
	Traditional healer	502	21.2
	Place of worship	62	2.6
	Other sources	322	13.6

† , Each category was a standalone question.

### Factors associated with willingness to take the COVID-19 vaccines

Overall, the prevalence of willingness to take the COVID-19 vaccines was 83.3%. All reported sociodemographic factors, the perceptions of vaccine safety and effectiveness, the perception that one was at low risk of COVID-19 infection and generally being against all vaccines were associated with the willingness to take the COVID-19 vaccines at the bivariate analysis (Table 4). However, only the perception that the COVID-19 vaccines are safe (aOR = 31.56, 95% confidence interval [CI]: 16.02–62.12 for affirmative agreement and aOR = 9.38, 95% CI: 5.40–16.30 for a somewhat agree) and that the vaccines are effective (aOR = 5.92, 95% CI: 2.87–12.19 for affirmative agree and aOR = 2.90, 95% CI: 1.48–5.65 for a somewhat agree) remained significantly associated with the willingness to take COVID-19 vaccines (Table 5).

**TABLE 4:** Bivariate analysis of factors associated with willingness to take COVID-19 vaccines.

Characteristic	Categories	Willing to take COVID-19 vaccines				*p* †
		No		Yes		
		*n*	%	*n*	%	
Age	18–29	290	18.4	1287	81.6	< 0.001
	30–49	92	16.1	478	83.9	-
	50 and above	13	6.1	200	93.9	-
	Total	395	16.7	1965	83.3	-
Gender	Male	151	18.2	677	81.8	0.138
	Female	243	16.1	1268	83.9	-
	Other	1	4.8	20	95.2	-
	Total	395	16.7	1965	83.3	-
Highest level of education	Up to grade 12	146	20.0	584	80.0	< 0.001
	Diploma and undergraduate degree	135	17.8	625	82.2	-
	Postgraduate degree	111	12.8	755	87.2	-
	Total	392	16.6	1964	83.4	-
University status (position)	Student	360	18.6	1573	81.4	< 0.001
	Professional staff	15	10.9	123	89.1	-
	Academic staff	10	4.2	226	95.8	-
	Services	9	18.4	40	81.6	-
	Total	394	16.7	1962	83.3	-
Perceiving COVID-19 vaccines are safe	Agree	31	2.7	1103	97.3	< 0.001
	Somewhat agree	113	13.4	728	86.6	-
	Somewhat disagree	127	56.2	99	43.8	-
	Disagree	123	78.3	34	21.7	-
	Total	394	16.7	1964	83.3	-
Perceiving COVID-19 vaccine are effective	Agree	69	4.7	1390	95.3	< 0.001
	Somewhat agree	168	25.1	501	74.9	-
	Somewhat disagree	70	57.9	51	42.1	-
	Disagree	86	80.4	21	19.6	-
	Total	393	16.7	1963	83.3	-
Perceiving risk of getting COVID-19 is low	No	362	16.3	1858	83.7	0.026
	Yes	33	23.6	107	76.4	-
	Total	395	16.7	1965	83.3	-
Being against vaccines in general	No	336	14.8	1932	85.2	< 0.001
	Yes	59	64.1	33	35.9	-
	Total	395	16.7	1965	83.3	-

COVID-19, coronavirus disease 2019.

† , *p*-value based on the Chi-Square test.

**TABLE 5:** Factors associated with willingness to take COVID-19 vaccines.

Characteristic	Categories	Willing to take COVID-19 vaccine			
		Yes	%†	Adjusted OR	95% CI
Age	18–29	1287	81.6	1.00	-
	30–49	478	83.9	1.20	0.78–1.83
	50 and above	200	93.9	1.99	0.88–4.51
Gender‡	Male	677	81.8	1.00	-
	Female	1268	83.9	1.28	0.96–1.73
Highest level of education	Up to grade 12	584	80.0	1.00	-
	Diploma and undergraduate degree	625	82.2	1.27	0.91–1.79
	Postgraduate degree	755	87.2	1.06	0.71–1.60
University status (position)	Student	1573	81.4	1.53	0.56–4.18
	Professional staff	123	89.1	3.60*	1.10–11.75
	Academic staff	226	95.8	2.82	0.83–9.63
	Services	40	81.6	1.00	-
Perceiving COVID-19 vaccines are safe***	Agree	1103	97.3	31.56***	16.04–62.12
	Somewhat agree	728	86.6	9.38***	5.40–16.30
	Somewhat disagree	99	43.8	1.87*	1.07–3.26
	Disagree	34	21.7	1.00	-
Perceiving COVID-19 vaccine are effective***	Agree	1390	95.3	5.92***	2.87–12.19
	Somewhat agree	501	74.9	2.90**	1.48–5.65
	Somewhat disagree	51	42.1	1.83	0.88–3.80
	Disagree	21	19.6	1.00	-
Perceiving risk of getting COVID-19 is low	No	1858	83.7	1.00	-
	Yes	107	76.4	0.85	0.50–1.43
Being against vaccines in general	No	1932	85.2	1.00	-
	Yes	33	35.9	0.62	0.35–1.09

COVID-19, coronavirus disease 2019; OR, odds ratio; CI, confidence interval.

* , *p* < 0.05;

** , *p* < 0.01;

*** , *p* < 0.001.

† , Row percentage of ‘Yes’ responses across each category;

‡ , excluded the other gender because of the small sample.

## Discussion

Key findings of this study were that more than 80.0% of participants declared their willingness to be vaccinated. They also had a clear preference for Pfizer and Johnson and Johnson vaccines. Mainstream media and social media were sources of information and safety, and efficacy were prominent determinants of willingness to vaccinate. The trusted sources of information on COVID-19 vaccines were the general practitioners (40.6%), specialists (19.2%).

The findings of this study are consistent with the result of the Coronavirus Rapid Mobile Survey,^20^ which found, in May 2021, that 76.0% of adults in South Africa were willing to take the COVID-19 vaccine. In a global survey of acceptance of the COVID-19 vaccine, China had the highest positive responses (86.0%), Russia had the lowest positive responses (54.9%). South Africa (81.58%) fell in the group of the high tendency towards acceptance in middle-income countries, together with Brazil and India.^21^

Several studies have found a three-way relationship between the factors of perceived severity, perceived susceptibility and perceived benefits, which influenced the willingness to take the COVID-19 vaccine.^2,21,22,23^ Although most of the study population felt these vaccines were safe and effective, the concerns around side effects and the rapid clinical trials to test these vaccines are possible barriers; the primary motivation to take the vaccine included personal well-being, as well as the well-being of others. Thus, these factors meet two criteria of the triad (perceived susceptibility and perceived benefits) and will influence their willingness to take the vaccine.

The willingness may also have been influenced because the survey included participants in a tertiary education facility with better access to information and developed reasoning skills. A study in Thailand showed that increased health education and increased willingness in the uptake of influenza vaccinations,^24^ whilst a study in America suggested that people who were more knowledgeable about herd immunity, were also more willing to take vaccinations.^25^ A study in Nigeria found a high level of hesitancy amongst medical and nursing students, however marginally more under nurses, who had lower risk perception and knowledge of COVID-19.^26^

Vaccine hesitancy is an important hurdle in the willingness to be vaccinated, thus hesitancy is an essential component to address for a successful vaccination roll-out.^27^ Razia and colleagues^28^ proposed a 5-C model to influence vaccine hesitancy. This model consists of:

Confidence (importance, safety and efficacy of vaccines); Complacency (perception of low risk and low disease severity); Convenience (access issues dependent on the context, time and specific vaccine being offered); Communications (sources of information); and Context (sociodemographic characteristics).

In this study, although the main sources of health information concerning the COVID-19 vaccines were obtained via the department of health, social media (Facebook, Twitter, Instagram), mainstream media (radio, television and newspapers), and the medical aids, the most trusted source of the health information was from general practitioners and followed by specialists. This is new information in the South African context. Although many study participants obtained their vaccine-related information from social media, the study participants based in a tertiary institution seem discerning in interpreting the information and not be swayed by the conspiracy theories that abound on social media.^10^

The trusted source of information result is also in keeping with Opel et al.,^29^ who found that a child’s healthcare provider is a trusted resource in the parental decision to vaccinate their child. In vaccine-hesitant parents living in the United States, they cite the child’s healthcare provider as the key to changing their minds.

Most of the study population were keen on obtaining two types of COVID-19 vaccines currently offered in SA. Willingness to vaccinate may decrease if the other vaccine products become available in the market and people do not have a choice of the vaccine they will receive.

In countries where the COVID-19 vaccination has been successfully rolled-out to the majority of the population, primary healthcare and community-based teams played a central role in rolling out the vaccination programme.^30,31,32^ In this study, to increase the willingness to be vaccinated, most people preferred being vaccinated at their local pharmacy and their general practitioner, as opposed to large vaccination sites, which currently exist.

The findings of this study should be interpreted considering the following limitations. Firstly, a poor response rate (5%) introduces the selection bias of participation of those using the university email, internet access (for those working from home) and those interested in the topic. Secondly, a potential risk of a social desirability response of over-reporting willingness to take the COVID-19 vaccines but the online anonymous survey would have mitigated this bias. Thirdly, even though the university communitycomprises individuals from all walks of life and geographical areas in the Gauteng province, the findings may not be entirely generalisable to the general population but university communities in South Africa. Finally, because of the cross-sectional nature of the study, we are only able to determine the factors associated with willingness to take the COVID-19 vaccine.

## Conclusion

This study identified that the safety and efficacy of the COVID-19 vaccines are significantly associated with the willingness to be vaccinated against COVID-19. There is an urgent need to reinforce the communication around the safety of vaccines and effectiveness for the individual and the community, through doctors, media and other approaches to reduce vaccine hesitancy and increase the willingness to be vaccinated. The policymakers should consider incorporating the community pharmacies and the GPs in the primary care-led vaccination roll-out, in addition to the current large vaccination sites, to achieve herd immunity as soon as possible.
